# History in context: teaching the history of dentistry with rare materials

**DOI:** 10.5195/jmla.2024.1934

**Published:** 2024-10-01

**Authors:** Nicole Theis-Mahon, Anna Opryszko, AI Miller, Emily Beck, Lois Hendrickson

**Affiliations:** 1 theis025@umn.edu, Associate Librarian, Health Sciences Library, University of Minnesota, Minneapolis, MN; 2 oprys002@umn.edu, Assistant Curator, Wangensteen Historical Library of Biology and Medicine, University of Minnesota, Minneapolis, MN; 3 aimiller@umn.edu, Reference Specialist, Health Sciences Library, University of Minnesota, Minneapolis, MN; 4 ebeck@umn.edu, Associate Curator, Wangensteen Historical Library of Biology and Medicine, University of Minnesota, Minneapolis, MN; 5 l-hend@umn.edu, Curator, Wangensteen Historical Library of Biology and Medicine, University of Minnesota, Minneapolis, MN

**Keywords:** History of Dentistry, Active Learning, Librarians, Curators, Health Humanities

## Abstract

**Background:**

History and health humanities instruction offers a framework for professional students to examine the arc and development of their profession as well as develop cultural competencies. Exploring ideas, themes, and health care practices and approaches through historical instruction can show students how culture influences health care and practice, therefore providing a context for further development of cultural competence skills.

**Case Presentation:**

This case report describes a collaboration among a dentistry course instructor, a liaison librarian, and curators of a rare book collection. Working together, this team offers an active learning class that examines the historical arc of the dental profession. We aim to have students use primary source materials to examine the experiences, research, and narratives of their profession.

**Discussion:**

Using a World Cafe with thematic tables allows students to safely examine rare materials and artifacts and have meaningful conversations about themes that are critical to dentistry’s past, present, and future. Students reported that engaging with artifacts and historical materials provided a different way to understand history and enhanced their learning experience. Engaging students in this work builds critical thinking skills that are essential to evidence-based practice.

## BACKGROUND

Contemporary health sciences curricula seek to train their students in systemic challenges within health care. Educating students about structural inequities is essential for promoting an understanding of how systemic biases in health science research, education, and access to health care can lead to disparate health outcomes. In dentistry and dental education, there is a move away from racebased to race-conscious care by efforts to recognize and reduce biases [1–2]. History holds lessons about how practices and beliefs like racial, sexual, and class-based biases have been embedded in the foundations and frameworks of the health sciences. Medical humanities and explorations of the history of dentistry session can highlight the continuities of those biases into present practice, create conversation about the foundations of dental practice, and raise critical questions of the source of the practices dental students would learn through their education [3].

Dentistry offers a unique opportunity in the context of health disparities and historical education. In the United States, separate systems for medical and oral health care have exacerbated disparities and increased barriers in access to oral health care [4]. Teaching the history of dentistry can provide a unique lens for understanding the professionalization of dentistry, its separation from medicine, and the impact of access to oral health care. Unfortunately, teaching the history of dentistry is not currently prioritized in the curriculum [5].

Historical medical libraries are widely viewed as unique and distinctive collections for facilitating student learning, as their primary resources can guide students through complex and challenging conversations related to the impacts of health care inequity that will remain relevant to them throughout their career as a health professional [6–7]. Institutions have integrated teaching with historical medical collections into medical education curricula to help students gain perspective and context about their scientific and clinical learning, and to understand that, as Barr et al. state, “medical practice is shaped by a multitude of social, cultural, and political forces” [8]. By working with materials that demand analytic engagement, students are presented with opportunities to grow in their critical thinking skills through interpretation and self-reflection [9].

As in other health sciences, integrating the range of knowledge, skills, and abilities required to effectively use historical primary resources (primary source literacy) into dental education activates critical thinking skills. Engaging students in this work can foster the development of practical skills that are essential to evidence-based practice, which prioritizes a rich continuum of experience, research, and narrative that professionals build, revisit, and reflect upon throughout their careers [3, 8, 10-12].

## CASE PRESENTATION

The Health Sciences Library (HSL) and Wangensteen Historical Library of Biology and Medicine (WHL) support courses and curriculum in the Academic Health Sciences at the University of Minnesota (UMN). A move to a new building (2021) presented an opportunity for both libraries to revisit their objective of increasing collaboration between the HSL liaisons and WHL curators. To facilitate this the WHL curators hosted subject-specific tours for liaisons showing the breadth of materials (print and artifact) related to their subject. The liaison librarian to the School of Dentistry participated in a tour and left with knowledge of materials relevant to dentistry and an invitation to seek out opportunities to collaboratively support courses and students in the School of Dentistry.

In March 2022, the liaison librarian met with an international cohort of dental students from Germany to tour library spaces and discuss services offered by the HSL. This also included a visit to the WHL where the students explored a curated set of historical dental materials which resulted in a thoughtful conversation around the history of dentistry and intersections with modern dental education and practices. The UMN coordinator of the international program was impressed with the ways students engaged with the materials and asked the liaison librarian and WHL curators to develop a similar session for a history of dentistry class session for year one dental students. The liaison librarian, curator, associate curator, public services supervisor, reference specialist, and course director formed a group (henceforth referred to as the “project team”) to develop a course drawing broadly on their pedagogical, subject content, and historical expertise.

The project team developed a two-hour session that is part of Dental Professional Development, a required course for year one Doctor of Dental Surgery (DDS) students (n=105). The project team’s objective was for students to contextualize the history of dentistry through rare books and artifacts, as well as to analyze materials with the goal of understanding dentistry’s past so they could better understand its future.

The course director shared curricular content from prior classes to provide context. The project team reviewed the materials, electing to build an assignment around Jane A. Weintraub’s “What Should Oral Health Professionals Know in 2040: Executive Summary,” which projected future trends and developments for dentistry [13]. The project team identified five themes from Weintraub’s article that connected dentistry’s past, present, and future that would become the framework for the class: (1) the connection between oral and systemic health; (2) diversity, equity, and inclusion; (3) aesthetics; (4) technology; and (5) patient consumerism for oral health care [3]. These themes are significant because they connect to evidence-based practice and current conversations that the students will be engaged in throughout their dental education.

The project team followed the 2021 revisions to Bloom’s taxonomy as they developed content for the course [14]. To provide a foundation for the course session, the project team created an ArcGIS StoryMap, “History of Dentistry in Context” [15]. It includes a timeline of major events in the history of dentistry, selected popular culture highlights to situate students in the historical framework being discussed, and deep-dive sections on the five thematic areas to prompt students to think about how culture shapes health, health care, and the experience of practitioners.

The curators used their expertise to select and finalize a group of historical primary sources that reinforced the themes. A learning approach that prioritizes skill development in historical and primary source analysis and critical thinking is foundational to teaching in the WHL library [16]. Artifacts and texts are not presented as oddities or curios, but as materials that should be interrogated and contextualized with other sources to understand both historical and contemporary people, cultures, events, and practices. This active learning approach aims to humanize history where students can think about the context of an item, how it was used, the intent behind a text or idea, and connect it with their present situation. The challenge that this class presented was how to structure it so 105 students could have meaningful interactions with rare materials in an active way.

A World Cafe structure was proposed for the class. Originally seventy items were identified as potentially relevant to the course. The project team reviewed each item, identified and bookmarked excerpts related to the themes, and chose which thematic area each item would go in. Content selected for the course was in English, with a focus on dentistry in the United States, and covered roughly the mid-1800s to early 1900s with most materials from the 1920s (Appendix A). These materials were chosen since the students were primarily English speakers from the United States and we wanted materials they could easily comprehend, and which may seem familiar during their dental education. The project team also chose a small selection of historical dental artifacts for the students to peruse before and after class. We organized the final selection of sixty items among the five themes with each theme having two tables. The students were divided into groups of ten to twelve people and rotated through the five thematic tables. The project team wrote three to four questions per theme to engage students with the materials, prompt exploration and discussion about the theme, and consider how the history of dentistry is related to the present and future of dentistry (Appendix B). Student groups were invited to respond to these questions on large Post-it Notes at each table and encouraged to build upon one another's comments. Facilitators were also at each table to prompt discussion and inquiry among the students. Each group spent about fifteen minutes at each table before rotating to the next theme.

At the end of the course session there was a debrief with the class where students shared what they found surprising or what they learned as they examined, thought about, reflected on, and discussed the rare materials and questions. Students shared feedback and their reflection of the class via an optional Qualtrics form (Appendix C). The Qualtrics questionnaire was submitted to the UMN Institutional Review Board and granted exempt status (STUDY00019894). The form consisted of seven required Likert scale questions and two open-ended questions which asked:

- What is one way that you think the themes presented today apply to your education and future as a dental practitioner? (required)
- Please share feedback or suggestions for future history of dentistry sessions. (optional)

A copy of the Qualtrics questionnaire can be found in the supplemental materials (Appendix C).

### Impact of teaching with historical materials and artifacts

The Qualtrics questionnaire had a response rate of 59.05% (n=62). Students indicated that their interest in the history of dentistry was stimulated by this session. Most respondents indicated that they learned something new, that the StoryMap was useful, and that they were planning to tell someone about the session (Table 1).

**Table 1:** Responses to History of Dentistry Questionnaire

	Strongly or Somewhat Agree (%)	Neither (%)	Strongly or Somewhat Disagree (%)
Learned something new	61 (98.39)	1 (1.16)	–
Enjoyed working with artifacts and historical texts	59 (95.16)	2 (3.23)	1 (1.16)
Engaging with historical texts/artifacts provided a different way to learn	60 (96.77)	2 (3.23)	–
Working with artifacts/historical texts enhanced my learning	61 (98.39)	1 (1.61)	–
The StoryMap provided historical context relevant to the history of dentistry	58 (93.55)	3 (4.84)	1 (1.61)
	**Yes (%)**	**Might (%)**	**No (%)**
I'm planning to tell someone about the things I learned today.	53 (85.48)	7 (11.29)	2 (3.23)

An open-ended question at the end of the Qualtrics questionnaire asked: What is one way that you think the themes presented today apply to your education and future as a dental practitioner? A thematic analysis was conducted of the responses to identify how respondents connected class content to their education and future as a dental practitioner. Two main themes emerged: (1) history as a linear trajectory, and (2) growth of the dental profession.

The first theme, history as a linear trajectory, is a progressive view of history where the past is bad, and the future is good. Students commented that “It was good to learn the mistakes of the past, so that we don't repeat them in the future,” and “We saw a lot of outdated attitude[s] towards dentistry and even what would now consider as malpractice today in dentistry.” This contrasts with a second perspective theme, which appreciates the intersection of the profession’s past with its present and recognizes the past as the foundation upon which the present and future are built. Students were able to juxtapose dentistry’s past and present, “What we learned today shows how much dentistry has advanced and change[d] throughout time, which makes you realize that it could change in our lifetime as well.” Students also identified themes from the past that continue to be relevant today, “Some concepts from older books that discussed things such as the systemic and oral health connections stay true today though” and recognized that, “Past dentistry built a solid foundation for today[‘s] dentistry and has been used as inspiration for modern dental practices.”

## DISCUSSION

Collaboration between a liaison librarian, curators, and course director resulted in a unique, engaging, and impactful class. The active, hands-on session allowed students to engage with major themes and questions that have impacted dentistry’s past and future. Students were engaged throughout the two-hour class and students had active conversations about the materials and the themes. Using a World Cafe provided a structure for students to move throughout the classroom and learn about the five thematic areas. Students were more interested in artifacts, such as dental instruments and tools, which was expected. With a one-shot class it can be challenging to engage students with print and physical materials that they are seeing for the first time but identifying specific excerpts and giving students a few minutes to explore materials allows for engaging conversations at the tables. Facilitators also assisted in prompting conversation by asking questions related to the theme and materials. The StoryMap provided valuable content and a grounding in historical thoughts, events, and developments in dentistry and dental care. The combination of brief narrative text, images, and timeline allowed for students to comprehend when themes were emerging in dentistry as well as the societal and cultural events of the time that were influencing dentistry. Several students referred to the content of the StoryMap in their facilitated in-class discussions.

Overall, students connected themes from dentistry’s past to dentistry’s present and were aware of its influence on today’s scientific thought. However, there is nuance in thinking about how history impacts the health sciences. Some students understood history as disconnected from present practices, relying on a historical narrative that emphasizes linear progress, noting that “I think it [history] applies to reflecting on mistakes and how they can be prevented in the future.” This can be viewed as a rupture from the past and creates a triumphalist narrative where present practices and thoughts overcame the biased science of the past [3]. Our intention for the class was to move students beyond this perspective so they can see the social dimensions, ideologies, and practices from the past that continue to influence dentistry today [3,11]. There is value in connecting with the “emotional aspects” of practice from today and the past [8]. It can be challenging to break from the view of linear progress, but some of the students started to understand this, indicating that “It is important to know about the past and how it impacts current dentistry of the day, and how we can make dentistry better in the future.” For future classes, the project team will edit questions for the facilitators to center table discussions on the growth of the dental profession, so students can identify and explore how the past continues to affect current dental practice.

While we were able to conduct this class in person with physical materials, it is possible to conduct these sessions virtually or with digital surrogates of historical sources. Online digital collections from archives and libraries offer opportunities for those who may not have access to an archival or rare book collection to engage with these materials for historical instruction [17]. During the COVID-19 pandemic, limitations in access to physical collections resulted in archival and special collections pivoting to use digital surrogates for instruction [18]. Having students engage with materials, either physical or digital, in small groups is needed for fostering meaningful discussions about the context of materials and topics [12]. Utilizing active learning techniques, such as a World Cafe, offers a structure for students to explore digital surrogates and have interactive conversations around specific topics or questions.

Co-creation of course material was essential to ensuring that students could see connections between complex historical material and their education as dental practitioners. While this case focused on historical instruction in dentistry, this kind of class session would be meaningful in other health sciences disciplines. Materials can be selected to cater to specific disciplinary needs. For example, a public health class may utilize materials and artifacts to discuss social determinants of health and community health needs [11], while a pharmacy class may explore changes in drugs and medicine to access to health care and changes to the role of the pharmacist [19].

Historical instruction is vital to professional programs, although it is a topic that is often overlooked and dismissed in favor of other requirements and the competition for space in the curriculum. It is a foundation for one’s professional identity. Thinking about perspectives, populations, or patients who are or are not represented in the historical record shows that professional identity and the evidence knowledge base may be on shakier ground than one initially thinks. Examining difficult or uncomfortable questions within a historical context can sometimes be easier for students since it allows them distance from the conversation. Learning about themes that are critical to understanding the past, present, and future provides students with an opportunity to explore wicked problems, such as the siloing of dentistry and impact of social determinants of health on dental care, and to think critically about them in a setting that fosters challenging questions and difficult conversations.

## Data Availability

Data associated with this article are available in the Open Science Framework at: http://dx.doi.org/10.17605/OSF.IO/AMVSJ.

## SUPPLEMENTAL FILES

**Appendix A:** Bibliography of Materials

**Appendix B:** Table Questions

**Appendix C:** Qualtrics Questions
