# Supplementary material for: History in context: teaching the history of dentistry with rare materials
Source: J Med Libr Assoc. 2024 Oct 7;112(4):372–7. doi: 10.5195/jmla.2024.1934 (PMC11486037; doi:10.5195/jmla.2024.1934)
Supplement: Supplementary file 1 — Appendix A: Bibliography of Materials [file jmla-112-4-372-s01.docx]

**Appendix A. Bibliography of materials**

Bibliography of books and other materials used for the thematic titles, for some of the books the library had multiple copies which were used for the class. We recognize that these materials are limited in scope (primarily United States and English language) and do not provide a global perspective.

*Oral and Systemic Health*

1. Stillman PR, McCall JO. A textbook of clinical periodontia: a study of the causes and pathology of periodontal disease and a consideration of its treatment. New York: The Macmillan company; 1922.
2. Tousey S. Roentgenographic diagnosis of dental infection in systemic diseases. New York: P.B. Hoeber; 1916.
3. Head J. Everyday mouth hygiene. Philadelphia: W.B. Saunders Company; 1920.
4. Nicholles J. The teeth, in relation to beauty, voice, and health: being the result of twenty years' practical experience and assiduous study, to produce the full development and perfect regularity of those essential organs. 2n
5. d ed. London: Hamilton, Adams; 1834.
6. Duke WW. Oral sepsis in its relation to systemic disease. St. Louis: Mosby; 1918.
7. Owre A. Diet and dentistry. Minneapolis: University of Minnesota; 1924.
8. Wallace JS. Dental diseases in relation to public health. London: The Dental Record; 1914.
9. Crocker AA. Modern dentistry for the laity and industrial dentistry for the corporation: modern preventive dentistry and industrial welfare dentistry. 3nd ed. Cincinnati: The Dental Register; 1920.
10. Price WA. Dental infections, oral and systemic. Cleveland, Ohio: The Penton publishing company; 1923.
11. Periodontia notes. Minneapolis; 1933.

*Diversity, Equity, and Inclusion (DEI)*

1. Fones AC. Mouth hygiene: a text-book for dental hygienists. 2nd ed. Philadelphia: Lea & Febiger; 1921.
2. Marshall JS. Mouth hygiene and mouth sepsis. 2nd, rev. ed. Philadelphia: J.B. Lippincott; 1916.
3. Hopkins SA. The care of the teeth. New York: D. Appleton; 1903.
4. Hecht CE. The gateway to health: prevention of diseases of the teeth. London: Pub. for the Food education society, by the St. Catharine press; 1921.
5. Ambler HL. Facts, fads and fancies about teeth. Cleveland: Helman-Taylor; 1900.
6. Talbot ES. The etiology of osseous deformities of the head, face, jaws and teeth. 3rd , revised and enlarg ed. Chicago: The W. T. Keener Company; 1894.
7. Wallace JS. Child welfare and the teachings of certain dentists, school medical officers, medical officers of health and other medical men. London: Baillière, Tindall and Cox; 1919.
8. Free Dental Inspection in Springfield, Mass. Dent Magazine. 1909 Jan; 4(1): 51-52.
9. Thomas, EA. Dentistry Relative to State Wards. Dent Magazine. 1909 Feb; 4(2): 114-118.

*Aesthetics*

1. Restoring the expression with artificial teeth: an explanation in pictures and words of how the expression of the face changes after the natural teeth are lost, and how it may be restored by the use of well selected artificial teeth. New York; 1916.
2. Prinz H. Dental materia medica and therapeutics: with special reference to the rational application of remedial measures to dental diseases; a textbook for students and practitioners. 5th, enl. and rev. ed. St. Louis: Mosby; 1920.
3. Ryan TJ, Bowers EF. Teeth and health: how to lengthen life and increase happiness by proper care. New York: G. P. Putnam's sons; 1921.
4. Prothero JH. Prosthetic dentistry. 2nd, rev. and enl. ed. Chicago: Medico-dental Publishing Co.; 1916.
5. Wallace JS. The teeth and health. London: Faber and Gwyer, ltd.; 1926
6. Morton WTG. On the loss of the teeth, and the modern way of restoring them. 2nd of the ten thousand copies. ed. Boston: Hall; 1848.
7. Eskell FA. A new system of treating and fixing artificial teeth: the art to prevent the loss of the teeth, with instructions calculated to enable heads of families to adopt the author's practice of treating and preserving the teeth. 3rd By Frederick A. Eskell. ed. Manchester: Printed for the author; 1862.
8. Clapp GW, Tench RW. Professional denture service. New York: The Dentists' supply company; 1918.
9. Morton WTG. On the loss of the teeth, and the modern way of restoring them. 2nd of the ten thousand copies. ed. Boston: Hall; 1848.

*Technology and Dental Treatments*

1. Miller WD. The micro-organisms of the human mouth: the local and general diseases which are caused by them. Philadelphia: White Dental Mfg. Co.; 1890.
2. Bruck WW, Jenkins CW. The filling of teeth with porcelain (Jenkins's system): a text book for dentists and students. New York: Consolidated Dental Mfg. Co.; 1902.
3. Raper HR. Radiodontia (dental radiography and diagnosis) questions and answers. Brooklyn, N.Y: Dental items of interest publishing co.; [etc., etc.]; 1923.
4. Lennox RP. Some methods and appliances in operative and mechanical dentistry. London: C. Ash and Sons; 1897.
5. Jordan ME. Operative dentistry for children. Brooklyn, N.Y: Dental items of interest publishing co.; [etc., etc.]; 1925.
6. Hogeboom FE. Practical pedodontia, or juvenile operative dentistry and public health dentistry: an introductory text for students and practitioners of dentistry. St. Louis: The C. V. Mosby company; 1924.
7. Posner JJ. Local anesthesia simplified. St. Louis: Mosby; 1925.
8. Dental radiographic technique. Chicago: [publisher not identified]; 1923.
9. Illustrated and descriptive catalogue: classified into departments and numbers. New York: Consolidated Dental Mfg; 1897.
10. Thoma KH. Oral anaesthesia; local anaesthesia in the oral cavity: technique and practical application in the different branches of dentistry. Boston, Mass: Ritter and Flebbe; 1914.
11. Hassler WC. Interpretation of the Radiograph. Dent Topics. 1919 Dec; 4(10): 11-12.
12. The Psychological Factor in Novocain Anesthesia. Dent Topics. 1920 Oct; 5(8): 5-6.
13. Tooth Key [object]. [circa 1850-1900]. Located at: Wangensteen History Library of Biology and Medicine, University of Minnesota, Minneapolis, MN.
14. Throat mirror [object]: Germany. Located at: Wangensteen History Library of Biology and Medicine, University of Minnesota, Minneapolis, MN.
15. Case of dental instruments [object]. [circa 1880-1920]. Located at: Wangensteen History Library of Biology and Medicine, University of Minnesota, Minneapolis, MN.

*Patient Oral Healthcare and Consumerism*

1. Black GV. A work on special dental pathology devoted to the diseases and treatment of the investing tissues of the teeth and the dental pulp: including the sequelae of the death of the pulp; also, systemic effects of mouth infections, oral prophylaxis and mouth hygiene. Chicago: Medico-Dental Pub. Co.; 1915.
2. White JW. The mouth and the teeth. Philadelphia: Lindsay & Blakiston; 1879.
3. Zonite: the antiseptic that actually kills germs. New York: Zonite Products Co.; 1926.
4. Turner CE, Rice W. Hygiene, dental and general. St. Louis: C. V. Mosby; 1920.
5. Bell VC. Popular essays upon the care of the teeth and mouth. 10th, carefully rev. ed. New York: P.P. Simmons; 1912.
6. Warner DC. The practical family dentist: with a variety of useful receipts for remedial compounds, designed for diseases of the teeth and gums. New York: Fowlers and Wells; 1853.
7. Fones AC, Kirk EC, Strang RHW, Fones AC. Mouth hygiene: a course of instruction for dental hygienists, a text-book containing the fundamentals for prophylactic operators. Philadelphia; Lea & Febiger; 1916.
8. Child health library. New York, N.Y: Robert K. Haas; 1924.
9. Towne RS. Your teeth: a study in oral hygiene. Chicago, Ill: W. M. Welch manufacturing company; 1922.
10. Lehn and Fink Products Corporation Distribution. Pebeco tooth powder box [object]. [circa 1940-1950]. Bloomfield, IN. Located at: Wangensteen History Library of Biology and Medicine, University of Minnesota, Minneapolis, MN.
11. E.W. Hoyt and Co. Rubifoam liquid dentifrice for the teeth [object]. [circa 1880-1920]. Lowell, MA. Located at: Wangensteen History Library of Biology and Medicine, University of Minnesota, Minneapolis, MN.

Additional items of interest (not used for the thematic tables). These were available for students to view before class or during the break.

1. Fauchard P. Le chirurgien dentiste, ou, Traitee des dents : ou l'on enseigne les moyens de les entretenir propres &e saines, de les embellir, d'en réparer la perte &e de remédier à leurs maladies, à celles des gencives &e aux accidens qui peuvent survenir aux autres parties voisines des dents. Deuxieme edition revûë, corrigée &e considérablement augmentée. ed. A Paris: Chez Pierre-Jean Mariette et chez l'auteur; 1746.
2. Fox J. The natural history and diseases of the human teeth. 2nd, enl. ed. London: Cox; 1814.
3. Hunter J. The natural history of the human teeth. 2nd ed. London: J. Johnson; 1778.
4. Pewe M. Medical receipts. [circa 1637-1661]. 537 leaves. Located at: Wangensteen History Library of Biology and Medicine, University of Minnesota, Minneapolis, MN.
5. Cameron’s Surgical Specially. Cameron’s right angle dentalamp. [object]. [circa 1920-1930]. Chicago, IL. Located at: Wangensteen History Library of Biology and Medicine, University of Minnesota, Minneapolis, MN.
6. Dental blowpipe [object] metal. Located at: Wangensteen History Library of Biology and Medicine, University of Minnesota, Minneapolis, MN.
7. W. Eyer. Surgical and dental instruments. [object]. [circa 1880-1920]. New York, NY. Located at: Wangensteen History Library of Biology and Medicine, University of Minnesota, Minneapolis, MN.
8. Mark B. Smith. Obstinhaler [object] 1889. Located at: Wangensteen History Library of Biology and Medicine, University of Minnesota, Minneapolis, MN.
