# Supplementary material for: History in context: teaching the history of dentistry with rare materials
Source: J Med Libr Assoc. 2024 Oct 7;112(4):372–7. doi: 10.5195/jmla.2024.1934 (PMC11486037; doi:10.5195/jmla.2024.1934)
Supplement: Supplementary file 2 — Appendix B: Table Questions [file jmla-112-4-372-s02.docx]

**Appendix B. Table Questions**

*Oral and Systemic Health*

- How does systemic disease impact oral health? What do dentists need to be aware of?
- Where, or how, can and has medical services be integrated into oral health? Weintraub asks, does the profession need two models of dentists in the future: a surgical model and a medical-oriented model?
- What are some potential challenges or problems that need to be solved to integrate oral and overall health?
- What can you learn from the integration of dentistry and medicine in the late 1800s to early 1900s? What would apply to today’s practice and what is different?

*Diversity, Equity, and Inclusion (DEI)*

- How has equity impacted access to oral health care? What barriers or challenges from the past persist with the present?
- The ADA’s commitment to diversity, equity, and inclusions states, “... representation matters, and every member is provided intentional opportunities to make meaningful contributions. Diverse viewpoints and needs are heard, valued, and respected.”
  - As you look at these historical materials, who is missing, which voices and perspectives are silent?
- Why is social justice important for dentistry and practicing dentists?

*Aesthetics*

- Why do, and have, people alter their teeth for aesthetic reasons?
- What similarities are there with modern patient questions or concerns about dental aesthetics and those of the past?
- Think about past aesthetic practices and present. What is different or has changed?
- What influence have advances in materials had on dental aesthetics?

*Technology and Dental Treatments*

- Changes and availability to new technology such as 3D imaging, intraoral scanning, methods to restore enamel, etc. are changing dental practice.
  - What similarities are there with today’s practice and these resources?
- Advances in technology or treatments can present ethical questions or considerations.
  - What ethical questions have been raised about the use of technology in past, present, and future dental practice?
- What do you find surprising when looking at previous treatments or technology used in dentistry?

*Patient Oral Healthcare and Consumerism*

- What kinds of dental care do dentists and other oral health practitioners expect their patients to engage in at home? How have these expectations changed over time?
- Oral healthcare products have changed over time.
  - What changes do you notice and why do you think those changes occurred?
  - Consider scientific, technological, and social developments.
- Historians of health often agree that efficacy is culturally constructed: Your own particular context defines whether something “works,” and that context, thus, also defines our expectations of treatments and cures.
  - What are some ways you notice that expectations around dental treatments and remedies changed over time?
  - Why do you think they’ve changed in these ways?

**This question is shared at each table (not theme specific):**

Other comments, observations, questions, or anything else you would like to share.
