# Supplementary material for: History in context: teaching the history of dentistry with rare materials
Source: J Med Libr Assoc. 2024 Oct 7;112(4):372–7. doi: 10.5195/jmla.2024.1934 (PMC11486037; doi:10.5195/jmla.2024.1934)
Supplement: Supplementary file 3 — Appendix C: Qualtrics Questions [file jmla-112-4-372-s03.pdf]

1. How much did this session stimulate your interest in the themes and the history of dentistry?

- ○ None at all
- ○ A little
- ○ A moderate amount
- ○ A lot
- ○ A great deal

---

2. As a result of participating in this session and using historical materials I learned something new.

- ○ Strongly disagree
- ○ Somewhat disagree
- ○ Neither agree nor disagree
- ○ Somewhat agree
- ○ Strongly agree

---

3. I'm planning to tell someone about the things I learned today.

- ○ Definitely not
- ○ Probably not
- ○ Might or might not
- ○ Probably yes
- ○ Definitely yes

---

4. I enjoyed the time spent in class working with artifacts and historical texts.

- ◯ Strongly disagree
- ◯ Somewhat disagree
- ◯ Neither agree nor disagree
- ◯ Somewhat agree
- ◯ Strongly agree

---

5. Engaging with historical texts/artifacts during class provided a different way to learn and understand dentistry and its history.

- ◯ Strongly disagree
- ◯ Somewhat disagree
- ◯ Neither agree nor disagree
- ◯ Somewhat agree
- ◯ Strongly agree

---

6. Working with artifacts/historical texts enhanced my learning about the history of dentistry.

- ◯ Strongly disagree
- ◯ Somewhat disagree
- ◯ Neither agree nor disagree
- ◯ Somewhat agree
- ◯ Strongly agree

---

7. The StoryMap (pre-work) provided me with historical context relevant to the history of dentistry?

- ○ Strongly disagree
- ○ Somewhat disagree
- ○ Neither agree nor disagree
- ○ Somewhat agree
- ○ Strongly agree

Page Break

8. What is one way that you think the themes presented today apply to your education and future as dental practitioner?

____

____

____

____

____

9. Please share feedback or suggestions for future history of dentistry sessions. (optional)

____

____

____

____

____

End of Block: Default Question Block
